# Plasma membrane vesicles of human umbilical cord mesenchymal stem cells ameliorate acetaminophen-induced damage in HepG2 cells: a novel stem cell therapy

**DOI:** 10.1186/s13287-020-01738-z

**Published:** 2020-06-08

**Authors:** Mei-jia Lin, Shuang Li, Lu-jun Yang, Dan-yan Ye, Li-qun Xu, Xin Zhang, Ping-nan Sun, Chi-ju Wei

**Affiliations:** 1 Guangdong Provincial Key Laboratory of Marine Biotechnology, Institute of Marine Sciences, Shantou University, Shantou, 515063 Guangdong China; 2 Research Center for Translational Medicine, The Second Affiliated Hospital of Shantou University Medical College, Shantou, 515041 Guangdong China; 3 Laboratory of Molecular Cardiology, The First Affiliated Hospital of Shantou University Medical College, Shantou, 515041 Guangdong China; 4 Stem Cell Research Center, Shantou University Medical College, Shantou, 515041 Guangdong China

**Keywords:** Acute liver failure (ALF), Plasma membrane vesicles (PMVs), Human umbilical cord mesenchymal stem cells (hUCMSCs), Stem cell therapy, Acetaminophen (APAP)

## Abstract

**Background:**

Acetaminophen (APAP) overdose is the common cause of acute liver failure (ALF) due to the oxidative damage of multiple cellular components. This study aimed to investigate whether plasma membrane vesicles (PMVs) from human umbilical cord mesenchymal stem cells (hUCMSCs) could be exploited as a novel stem cell therapy for APAP-induced liver injury.

**Methods:**

PMVs from hUCMSCs were prepared with an improved procedure including a chemical enucleation step followed by a mechanical extrusion. PMVs of hUCMSCs were characterized and supplemented to hepatocyte cultures. Rescue of APAP-induced hepatocyte damage was evaluated.

**Results:**

The hUCMSCs displayed typical fibroblastic morphology and multipotency when cultivated under adipogenic, osteogenic, or chondrogenic conditions. PMVs of hUCMSCs maintained the stem cell phenotype, including the presence of CD13, CD29, CD44, CD73, and HLA-ABC, but the absence of CD45, CD117, CD31, CD34, and HLA-DR on the plasma membrane surface. RT-PCR and transcriptomic analyses showed that PMVs were similar to hUCMSCs in terms of mRNA profile, including the expression of stemness genes GATA4/5/6, Nanog, and Oct1/2/4. GO term analysis showed that the most prominent reduced transcripts in PMVs belong to integral membrane components, extracellular vesicular exosome, and extracellular matrix. Immunofluorescence labeling/staining and confocal microscopy assays showed that PMVs enclosed cellular organelles, including mitochondria, lysosomes, proteasomes, and endoplasmic reticula. Incorporation of the fusogenic VSV-G viral membrane glycoprotein stimulated the endosomal release of PMV contents into the cytoplasm. Further, the addition of PMVs and a mitochondrial-targeted antioxidant Mito-Tempo into cultures of APAP-treated HepG2 cells resulted in reduced cell death, enhanced viability, and increased mitochondrial membrane potential. Lastly, this study demonstrated that the redox state and activities of aminotransferases were restored in APAP-treated HepG2 cells.

**Conclusions:**

The results suggest that PMVs from hUCMSCs could be used as a novel stem cell therapy for the treatment of APAP-induced liver injury.

## Background

Acute liver failure (ALF) is a clinical syndrome characterized by icterus, coagulopathy, and encephalopathy due to a sudden decline in liver function, and acetaminophen (APAP) overdose is the leading cause of ALF in Western countries [1]. APAP is effective and safe when taken at low doses for fever or pain relief. However, excessive intake can cause hepatic injury and even leads to ALF in humans [2]. The primary cause of APAP-induced liver injury is the formation of a reactive metabolite *N*-acetyl-p-benzoquinone imine (NAPQI), which depletes glutathione and results in cytotoxic NAPQI protein adducts [3]. Therefore, the formation of cytotoxic protein adducts, especially in mitochondria, is generally agreed to be associated with the onset of ALF. The likely mechanisms involve the increase of inner mitochondrial membrane permeability, mitochondrial swelling, production of reactive oxygen species (ROS), and decrease in ATP synthesis, which eventually leads to cell necrosis [4]. *N*-acetylcysteine (NAC) is the only clinically used antidote, acts as a glutathione precursor, and is most effective when administered at the early time points to prevent protein binding of NAPQI. Therefore, NAC functions at the earliest stage of NAPQI-driven dysfunction and incurs a limited therapeutic window [5].

In recent years, a growing number of studies have shown that mesenchymal stem cells (MSCs) can effectively treat ALF. MSCs are multipotent stromal cells that can differentiate into osteoblasts, chondrocytes, myocytes, and hepatocyte-like cells [6, 7]. Preclinical and clinical evidence demonstrated that the therapeutic benefit of MSCs in various medical conditions could be attributed to their regenerative abilities, low immunogenicity, immunomodulatory properties, and paracrine effects [8–11]. The mechanism of MSC treatment for ALF is primarily focused on immunoregulation and differentiation of MSCs into hepatocyte-like cells [12].

Although MSCs present great potential for the treatment of a myriad of diseases, however, recent studies have suggested that MSCs also contribute to tumor pathogenesis by supporting tumor microenvironments, increasing tumor growth, and suppressing antitumor immune responses [13–15]. Besides, a fraction of transplanted MSCs die shortly after injection and secrete extracellular vesicles and apoptotic bodies [16]. Lastly, MSCs may transdifferentiate into non-desirable cell types [17], which could jeopardize the physiological functions of the target tissue.

We have established a cutting-edge technology for the production of plasma membrane vesicles (PMVs), which enclose the cytosol and cellular organelles but not the nucleus of Ad293 cells, by a simple mechanical extrusion [18]. Our previous study also demonstrated that PMVs from bone marrow-derived MSCs were able to deliver the content of the cytoplasm into the gastrocnemius muscle of mice [19]. The lack of nuclear DNA in PMVs could potentially reduce the risk of tumorigenesis from MSCs and genome damage by DNA random integration. More importantly, unlike traditional stem cell therapies, PMVs function directly via transferring healthy cytosol or organelles to replace damaged cellular components in target cells.

In this study, we investigated whether PMVs of hUCMSCs, which are highly similar to hUCMSCs in terms of cell surface markers, the transcript profile, and cellular organelles, could be exploited for novel stem cell therapy using APAP-induced damage in HepG2 cell model. PMVs were generated using an improved procedure of chemical enucleation combined with mechanical extrusion. Further, we used a fusogenic viral membrane glycoprotein VSV-G to facilitate the release of PMVs into target cells. The results supported our hypothesis that PMVs of hUCMSCs might represent a potential novel stem cell therapeutic strategy against APAP-induced liver injury.

## Materials and methods

### Materials

Biochemical reagents, antibodies, and plasmids were purchased from companies indicated as follows: *N*-acetyl-p-aminophenol (APAP, Weikeqi Biotech, Chengdu, China); MitoTracker-Green, JC-1, LysoTracker-Red, Hoechst 33342, and FITC-conjugated goat anti-rabbit IgG (Beyotime, Shanghai, China); TMRE (Solarbio, Beijing, China); cytochalasin B and colchicine (Meilun, Dalian, China); Calcein-AM (Genesion, Guangzhou, China); Mito-Tempo (Sigma, St Louis, MO); pLP-VSVG (Clontech, Mountain View, CA); polyclonal rabbit anti-proteasome 20S α5 and anti-calnexin antibodies (Biosynthesis, Lewisville, TX); alanine transaminase (ALT); and aspartate transaminase (AST), glutathione (GSH), and glutathione disulfide (GSSG) test kits (Nanjing Jiancheng Bioengineering Institute, Nanjing, China).

### Umbilical cords and amnion collection

The umbilical cords and amnion were obtained from healthy full-term pregnancies undergoing cesarean section in the Department of Obstetrics and Gynecology, The Second Affiliated Hospital of Shantou University Medical College. Informed consent was taken before parturition from participants, and the study was approved by the ethical committee of Shantou University Medical College (Shantou, China).

### Cultivation and expansion of hUCMSCs

Isolation and cultivation of hUCMSCs were performed in our translational medicine center, as previously described [20]. Briefly, the umbilical cords were collected from the operating room within 24 h after the cesarean section and were rinsed with a phosphate-buffered solution (PBS). After removing two arteries and a vein buried within the mucous connective tissue, the Wharton’s jelly was separated and cut into small pieces of 2–3 cm in a sterile container containing high-glucose Dulbecco’s modified Eagle’s medium (H-DMEM, Gibco-BRL, Carlsbad, CA). The explants were incubated for about 15–30 min until they settled at the bottom of 100-mm cell culture dishes. About 5 ml of fresh growth medium (H-DMEM containing 10% fetal bovine serum, 100 mg/ml penicillin, 100 mg/ml streptomycin, and 1 mg/ml amphotericin B) was carefully added, and the dishes were transferred to a humidified incubator maintained at 37 °C with an atmosphere of 5% CO_2_. After 5–7 days, sporadic fibroblast-like cells grew from the tissue edge. The cells were trypsinized and passaged at a 1:4 split when cultures reached 80–90% confluence. hUCMSCs of 4–11 passages were used in all experiments.

### Measurement of in vitro differentiation potentials of hUCMSCs

The adipogenic, osteogenic, and chondrogenic differentiation potentials of hUCMSCs were determined by incubating them under differentiation conditions according to the instructions provided by the company (Cyagen Biosciences, Suzhou, China). Briefly, after induction in adipogenic medium (HUXUC-90031) for 2 weeks, cells were fixed with 4% formaldehyde for 20 min at room temperature and stained with Oil Red O. For osteogenic induction, cells were induced in differentiation medium (HUXUC-90021) for 3 weeks, fixed with 4% formaldehyde, then stained with Alizarin Red. For chondrogenic differentiation, cells were induced in differentiation medium (HUXUC-9004) for 3 weeks, fixed with 4% formaldehyde, then stained with Alcian Blue. After washing twice with PBS, cells were photographed under a microscope equipped with a CCD camera (Eclipse TE 2000, Nikon, Japan).

### Preparation of PMVs from hUCMSCs by enucleation and extrusion

PMVs from hUCMSCs were prepared with an improved procedure reported previously, including a chemical enucleation step followed by a mechanical extrusion [18, 19]. Enucleation was performed according to a reported protocol with modifications. A Percoll gradient was generated as follows: 1.5 ml of 25%, 1.5 ml of 17%, 0.375 ml of 16%, 0.375 ml of 15%, and 1.5 ml of 12.5%. Each Percoll gradient was then supplemented with cytochalasin B and colchicine to a final concentration of 10 μg/ml. The solutions were layered into a Quick-Seal centrifuge tube (6 ml, Beckman, Brea, CA), which was then incubated for 6 to 18 h at 37 °C. Around 1 × 10^6^ hUCMSCs in 100 μl of PBS supplemented with 10 μg/ml cytochalasin B and colchicine were layered on top of the Percoll gradient, and subsequently centrifuged at 35,000*g* for 1 h in a 100Ti fixed angle rotor (Optima L-100K, Beckman, Brea, CA), which had been pre-warmed to 37 °C for 1 h. Percoll sediment formed at the bottom after centrifugation. A mixture of intact cells, microcells, enucleated cells, and vesicles could be found floating above the Percoll sediment. The mixture was collected and then loaded into a syringe, which was attached to a filter unit (Xin Ya, Shanghai, China). The plunger of the syringe was pushed slowly to squeeze the mixture through a 5-μm polycarbonate membrane (Merck Millipore, Darmstadt, Germany) on the filter. An additional 5 ml of the medium was loaded into the syringe and was slowly pushed through the filter. All the media were collected after extrusion and centrifuged at 1000 rpm for 20 min to collect PMVs.

### Characterization of surface markers on hUCMSCs and PMVs

Surface markers on hUCMSCs were analyzed by flow cytometry. After trypsinization, approximately 1 × 10^6^ cells were fixed with 4% paraformaldehyde for 20 min at room temperature. Collected cells were then incubated with indicated PE-conjugated antibodies CD13, CD29, CD31, CD34, CD44, CD45, CD73, CD117, HLA-ABC, and HLA-DR (eBioscience, Shanghai, China) at room temperature for 2 h. Control samples were incubated with PE-conjugated mouse IgG1 isotype antibodies. After incubation, cells were washed with PBS and centrifuged to remove unbound antibodies. Cells were resuspended in 1 ml PBS and analyzed by flow cytometry using the Accuri C6 cytometer (BD, Franklin Lakes, NJ). Surface markers on PMVs were measured by fluorescence staining. PMVs from hUCMSCs were adhered to a 35-mm glass-bottom dish (In Vitro Scientific, Sunnyvale, CA), fixed with 4% paraformaldehyde, and incubated with the above PE-conjugated antibodies at room temperature for 2 h. After washing, PMVs were examined and photographed under a confocal microscope (LSM 800 Meta, Carl Zeiss, Germany).

### RNA isolation and RT-PCR

Total RNA from hUCMSCs and PMVs were isolated for PCR amplification of GATA4/5/6, NANOG, OCT1/2/4A/4B, CD29, CD44, CD73, CD90, CD105, and beta-actin transcripts as reported [21]. Three micrograms of total RNA was used for reverse transcription using random primers (Takara, Japan) and M-MuLV reverse transcriptase (Toyobo, Japan) in a total volume of 25 μl. After reverse transcription, the cDNA was diluted with H_2_O (Dnase and Rnase free, Toyobo) into a volume of 100 μl, of which 2 μl was used for PCR amplification in a total volume of 25 μl. The PCR conditions were 2 min at 94 °C, then 35 cycles of 94 °C for 30 s, 50–65 °C for 30 s, 72 °C for 1 min, and a final extension for 5 min at 72 °C. The amplified PCR products were examined by electrophoresis in a 1% agarose gel.

### RNA extraction, library construction, and sequencing

Samples of hUCMSCs and PMVs were dissolved in TRIzol Reagent (Invitrogen, Carlsbad, CA) and sent to the Total Genomics Solution (TGS) company (Shenzhen, China) for transcriptomic analysis. The RNA quality of each sample was monitored on a 1.5% agarose gel. RNA concentration and integrity of each sample were further determined using an Agilent 2100 Bioanalyzer and ABI StepOnePlus Real-Time PCR System. An equal amount of RNA from a sample was used for library construction using the Illumina TruSeq RNA Sample Preparation Kit (Illumina). Briefly, mRNA was isolated from the RNA using poly-T oligo-attached magnetic beads, and then the purified mRNA was cleaved into small fragments, followed by first-strand cDNA synthesis with M-MuLV Reverse Transcriptase and random hexamer primers. The second-strand cDNA was further synthesized, and sequencing adapters were ligated to the products. cDNA fragments within the size range of 200–300 bp were selected on a 2% agarose gel and were subjected to PCR amplification for 15 cycles. The cDNA libraries were then sequenced on the Illumina HiSeq X-Ten platform with 125-bp paired-end sequencing reads. The raw sequence data were filtered by removing adaptor sequences, low-quality reads with more than 5% anonymous nucleotides (N), and 50% bases of quality value ≤ 5 by using hierarchical indexing for spliced alignment of transcripts (HISAT). All reads were mapped to human genome hg38 by Tophat2 with default parameters. The reads per kilobase per million mapped reads (RPKM) of the gene expression was calculated based on the GENCODE v23 annotation. All expressions were normalized by the quantile normalization method using the median.

### Examination of cellular organelles in PMVs

The hUCMSCs were stained with respective fluorescence dyes, including MitoTracker-Green, LysoTracker-Red, or JC-1 in PBS for 30 min before being used for PMV preparation. PMVs were then stained with Hoechst for 30 min, washed, and seeded into a 35-mm glass-bottom dish (In Vitro Scientific, Sunnyvale, CA). PMVs were then examined under a confocal microscope (LSM 800 Meta, Carl Zeiss, Germany). To detect the enclosure of proteasome or endoplasmic reticulum (ER), PMVs were adhered to a 35-mm glass-bottom dish, fixed with 4% paraformaldehyde, permeabilized with 0.2% Triton-X, and then incubated with polyclonal anti-proteasome 20S α5 or anti-calnexin antibody, respectively, at room temperature for 2 h. After further incubation with FITC-conjugated goat-anti-rabbit IgG, the sample was examined under a confocal microscope.

### Preparation of viral membrane glycoprotein VSV-G

Ad293 cells (Agilent, Santa Clara, CA) were seeded in 6-wells overnight and transfected with 2 μg/well of pLV-VSVG plasmid using the PolyJet reagent (SignaGen Laboratories, Ijamsville, MD) as per instruction manual. Cells were harvested at 48 h, resuspended in 0.5 ml media, and extruded through a 3-μm and then 0.8-μm membrane filter. Media were collected and used for the preparation of fusogenic PMVs.

### Measurement of phagocytosis and endosomal escape of PMVs

hUCMSCs cells were stained with Calcein-AM (2 μM) for 30 min or MitoTracker-Green (200 nM) for 1 h before being used for PMV generation. VSV-G viral membrane glycoprotein was prepared as described. PMVs were incubated with or without VSV-G for 20 min and then added to HepG2 cell culture in 96-wells. The plate was centrifuged at 1000 rpm for 10 min to accelerate PMV sedimentation. At 12 h, cells were replenished with fresh culture medium. At 24 h, cells were harvested, stained with Hoechst (10 μg/ml) for 30 min, and then transferred to a 35-mm glass-bottom dish for the examination of phagocytosis and endosomal escape of PMVs using confocal microscopy.

### Preparation of ECM of HTB9 cells

Extracellular matrix (ECM) of HTB9 cells (Tongpai Biotech, Shanghai, China) was prepared as described in the previous study [22]. Briefly, HTB9 cells were propagated in DMEM supplemented with 10% fetal bovine serum (HyCLONE, Logan, UT), 2 mM glutamine, 50 U/ml of penicillin G sodium, and 50 mg/ml of streptomycin sulfate (Beyotime, Haimen, China). About 2 × 10^4^/well of HTB9 cells were seeded in a 96-well (Corning, NY, USA) and grew to 80–90% confluency after 48 h of cultivation. ECM was then prepared by lysis with a 20-mM ammonium hydroxide solution. The well was washed twice with 200 μl of de-ionized H_2_O, followed by washing twice with 200 μl of PBS. The cell culture plate with ECM was used immediately or stored in a refrigerator for up to 1 week.

### Co-culture of PMVs with APAP-treated HepG2 cells

About 2 × 10^5^ cells of HepG2 (ATCC, Manassas, VA) were treated with 90 mM APAP in 200 μl culture medium for 3 h. The medium was then discarded and washed with culture medium four times. 1 × 10^4^ cells were re-seeded in a 96-well precoated with ECM of HTB9 cells with or without 50 μM Mito-Tempo (MT). At 4 h, PMVs of hUCMSCs (with VSV-G) were added. PBS was used as a negative control. After 12 h of cultivation, cells were replenished with fresh culture medium with or without 50 μM MT. At 48 h, cells were stained with Calcein-AM (2 μM) and Hoechst (10 μg/ml) for 30 min to measure cell viability, or stained with TMRE (100 nM) and Hoechst (10 μg/ml) for 30 min to measure mitochondrial membrane potential by confocal microscopy. After that, cells were harvested and put into a 35-mm glass-bottom dish and analyzed again by confocal microscopy.

### APAP-induced HepG2 cell death analysis

APAP-treated HepG2 cells were incubated with Mito-Tempo or PMVs, as described before. PBS was used as a negative control. At 48 h, cells were stained with Annexin V-FITC (50 μg/ml), propidium iodide (100 μg/ml), and Hoechst (10 μg/ml) and subsequently analyzed by confocal microscopy.

### Measurement of intracellular biochemical indexes

As stated earlier, APAP-treated HepG2 cells were incubated with Mito-Tempo or PMVs. Intracellular levels of AST, ALT, total GSH, and GSSG were measured using respective assay kits (Nanjing Jiancheng Bioengineering Institute, Nanjing, China) according to the manufacturer’s instructions. Absorbance was measured at 510 nm or 405 nm in a 96-well plate using a Synergy H1 microplate reader (Biotek, Winooski, VT). Samples and standards were assayed three times in triplicate. GSH was calculated by subtracting 2-fold of GSSG from total GSH, and then the ratio of GSH to GSSG could be calculated.

### Statistical analysis

All statistical analyses were performed using GraphPad Prizm 7 (GraphPad Software Inc., San Diego, CA). All data represent the means ± standard deviation. The results were subjected to two-tailed Student’s *t* test and one-way ANOVA with Tukey’s post hoc test. *P* values < 0.05 were considered statistically significant.

## Results

### Characterization of hUCMSCs and PMVs

**Fig. 1:**
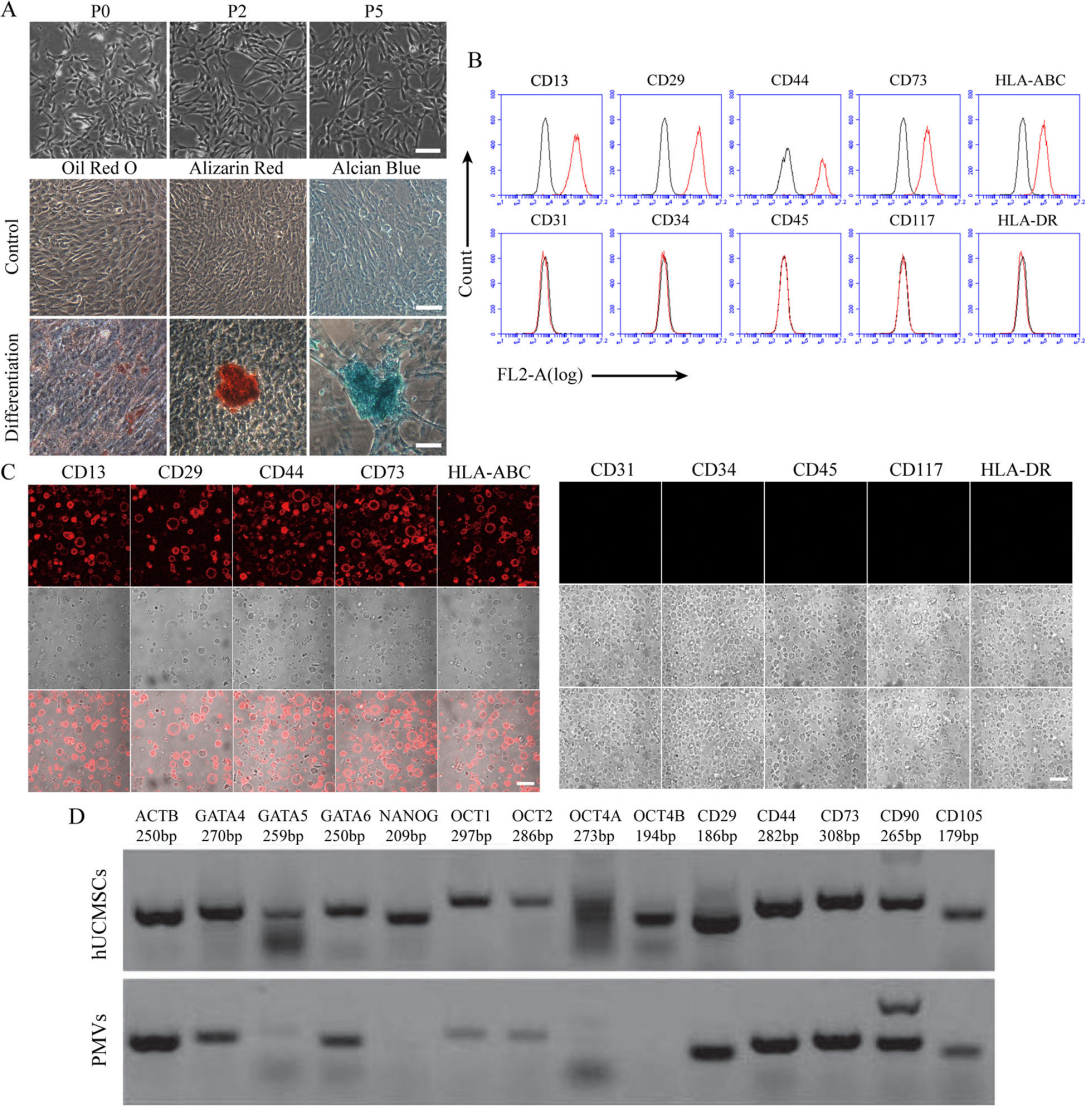
Characterization of hUCMSCs and PMVs*.***a** Microphotographs show cells of passages 1, 2, and 5 (upper panel). Passage 4 hUCMSCs were induced under adipogenic, osteogenic, and chondrogenic conditions for 2–3 weeks, and undifferentiated and differentiated of hUCMSCs were examined by Oil Red O, Alizarin Red, and Alcian Blue staining, respectively (lower panel), scale bar = 50 μm. **b** hUCMSCs were incubated with indicated PE-conjugated antibodies and then analyzed by flow cytometry; *n* = 3. An isotype-match antibody was used as a negative control. **c** PMVs were generated from hUCMSCs after staining with respective fluorescence-conjugated antibodies and then examined by confocal microscopy, scale bar = 10 μm. **d** Total RNA was harvested from hUCMSCs and PMVs. Transcripts of genes characteristic of hUCMSCs were amplified by RT-PCR and then analyzed by electrophoresis. The experiment had been repeated at least three times

Primary cultures of hUCMSCs were routinely established in our lab. Isolated hUCMSCs exhibited a typical fibroblast-like morphology (Fig. 1a). After cultivation under induced conditions for 4 days, hUCMSCs displayed adipogenic, osteogenic, and chondrogenic differentiation potential as demonstrated by Oil Red O, Alizarin Red, and Alcian Blue staining, respectively. Surface markers of hUCMSCs were then analyzed by flow cytometry, showing that hUCMSCs were positive for CD13, CD29, CD44, and CD73 but negative for CD45, CD117, CD31, CD34, and HLA-DR (Fig. 1b). The characteristic of hUCMSCs was assayed further by RT-PCR. The transcripts of surface markers (CD29, CD44, CD73, CD90, and CD105) and stemness genes (GATA4/5/6, NANOG, OCT1/2/4A/4B) typically expressed in hUCMSCs were detected (Fig. 1d).

Recently, we reported the generation of plasma membrane vesicles (PMVs) as a novel approach to deliver cellular proteins and organelles into target cells [18]. With a modified procedure, we produced micro-scale PMVs from hUCMSCs by chemical enucleation followed by extrusion through a membrane filter. Surface markers of hUCMSCs, including CD13, CD29, CD44, and CD73, were detected in PMVs by confocal microscopy (Fig. 1c), while CD45, CD117, CD31, CD34, and HLA-DR were not detectable on PMVs. Furthermore, transcripts of surface markers and stemness genes of hUCMSCs were mostly detected in PMVs (Fig. 1d), except that of GATA5, NANOG, and OCT4A/4B, probably due to low levels of expression.

### PMVs had a similar transcript profile as hUCMSCs

**Fig. 2:**
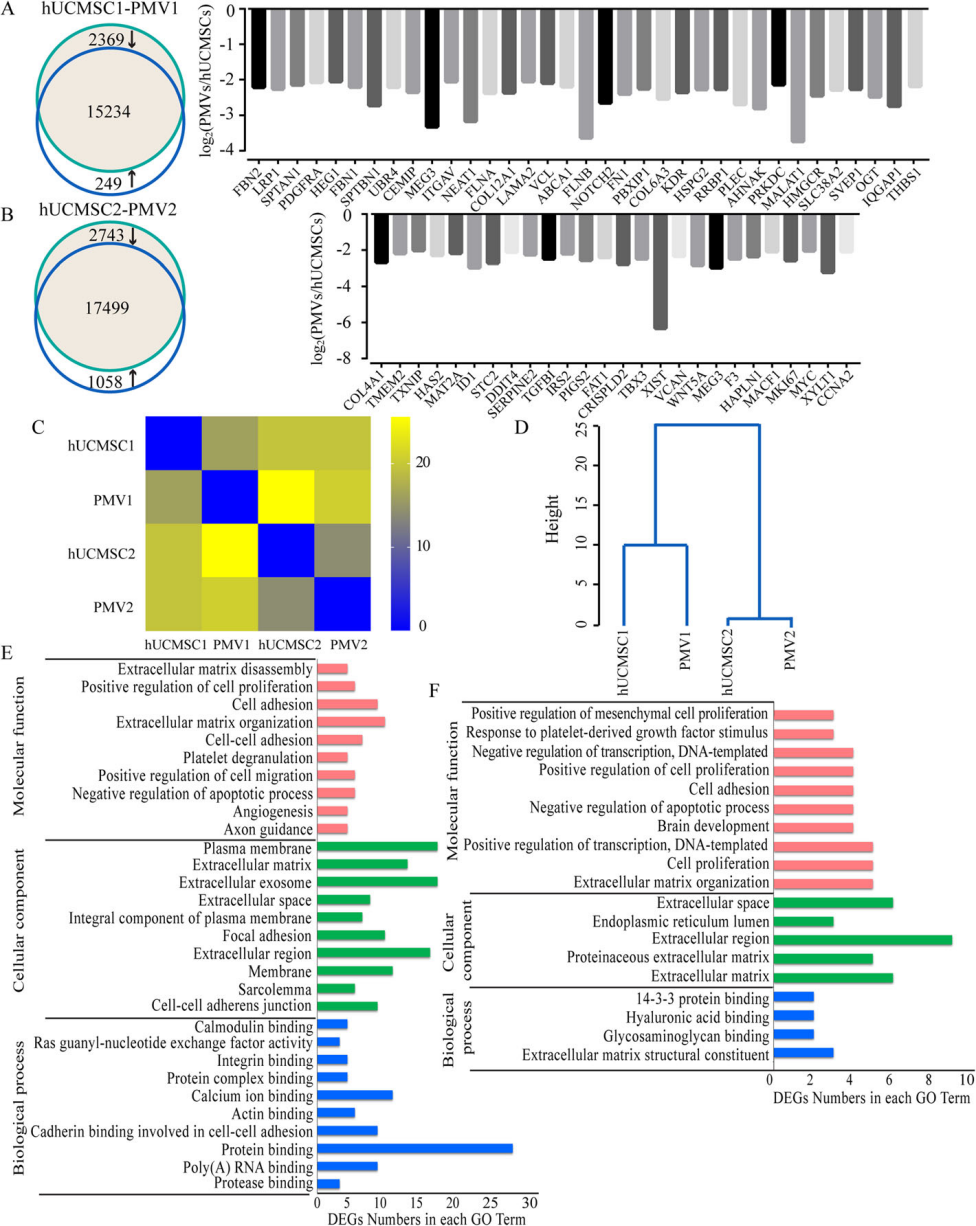
Bioinformatic analysis of the transcript profile of hUCMSCs and PMVs. Total RNAs were harvested from two preparations of hUCMSCs and PMVs and sent to Hengchuangjiyin Technology (China) for bioinformatic analysis (**a**, **b**) Diagrams (left) show the number of differentially expressed genes (DEGs) from preparations 1 and 2 of hUCMSCs vs. PMVs. The overlap of 2 circles represents common genes in both PMVs and hUCMSCs. Downregulated and upregulated DEGs were indicated with an arrow. Bar graphs (right) show genes with at least a 4-fold difference in the transcript levels for hUCMSCs vs. PMVs. The heat map of Euclidean distance (**c**) and hierarchical clustering profile (**d**) show the similarity between the transcripts of hUCMSCs and PMVs. The vertical distance reflects the similarity between samples (**c**). **e**, **f** GO term analysis of DEGs with at least 4-fold difference in the transcript levels between hUCMSCs and PMVs of preparations 1 and 2

To further compare the similarity between hUCMSCs and PMVs, total RNAs were harvested, and the transcript profile of mRNAs was analyzed. Among 17,852/21,300 genes from studies of two preparations (hUCMSCs1/PMV1, hUCMSCs2/PMV2) with at least one read count per million (RPKM ≥ 1), a total of 2369/2743 genes decreased, and 249/1058 genes increased for at least 2-fold in PMVs as compared to that of hUCMSCs (Fig. 2a, b). Among these, transcripts of the genes with at least 4-fold difference (35/26) were all downregulated in PMVs. Further, GO term analysis revealed that differentially expressed genes (DEGs) with 4-fold differences mostly belong to integral membrane components, extracellular vesicular exosome, and extracellular matrix of the cellular component categories, or cell adhesion, migration, and extracellular matrix organization of the biological processes, or protein binding and binding of other molecules of the molecular functions (Fig. 2e, f), suggesting that mRNAs that associate with ribosomes and bind to the endoplasmic reticulum (ER) were preferentially excluded from PMVs during the enucleation/extrusion processes. Besides, we constructed a pairwise similarity heat map based on Euclidean distance (Fig. 2c), which demonstrated a substantial similarity between hUCMSCs1 and PMV1, or hUCMSCs and PMVs. The conclusion was further confirmed by hierarchical clustering analysis (Fig. 2d), which also revealed a relatively bigger difference between two batches of hUCMSC preparations.

### PMVs of hUCMSCs enclosed cellular organelles

**Fig. 3:**
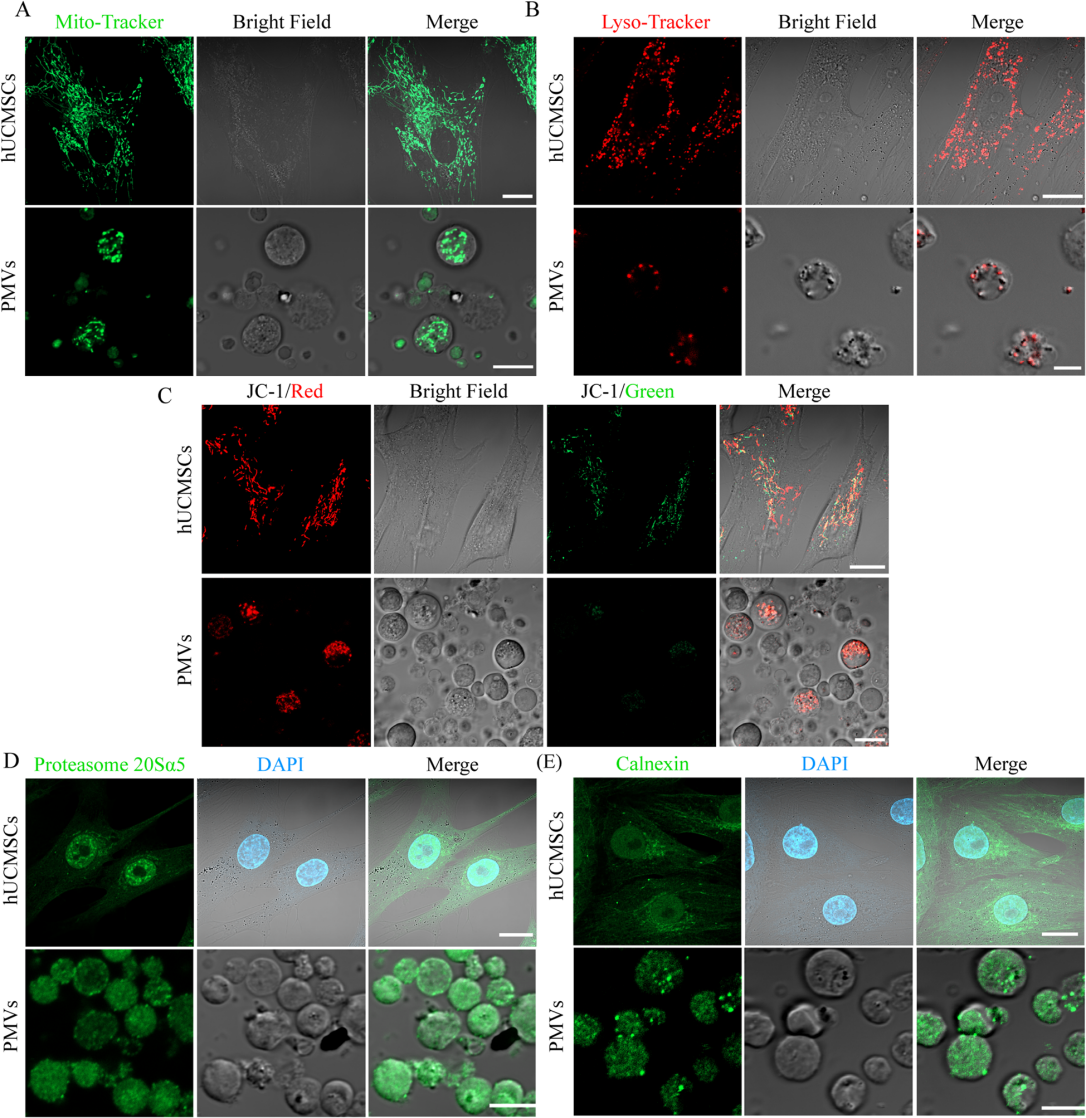
Detection of cellular organelles in hUCMSCs and PMVs by confocal microscopy. **a**–**c** Microphotographs show MitoTracker-Green (**a**, mitochondria), LysoTracker-Red (**b**, lysosome), and JC-1 (**c**, mitochondrial membrane potential) staining of hUCMSCs (upper panel, scale bar = 20 μm) and PMVs (lower panel, scale bar = 5 μm). **d**, **e** Microphotographs show the proteasome (**d**) and the endoplasmic reticulum (**e**) in hUCMSCs (upper panel, scale bar = 20 μm) and PMVs (lower panel, scale bar = 5 μm) detected by immunofluorescence staining for proteasome 20Sα5 (green) and calnexin (green), respectively. The images were taken by confocal microscopy after DAPI staining (blue)

The unique feature of PMVs associated with their micrometer size is the encapsulation of cellular organelles. In PMVs of hUCMSCs, mitochondria revealed by MitoTracker-Green staining were easily detected (Fig. 3a). Although a small portion of PMVs contained the nucleus when a 5-μm membrane was used for the extrusion, no Hoechst staining was detected after passing through a 3-μm membrane (data not shown). The mitochondria were functional as evidenced by JC-1 staining showing an intense red vs. green fluorescence (Fig. 3c). Further, the enclosure of the lysosome in PMVs was demonstrated by LysoTracker-Red staining (Fig. 3b). Lastly, the enclosure of proteasome and ER in PMVs was confirmed by immunofluorescence staining with antibodies against proteasome 20s alpha 5 and calnexin, respectively (Fig. 3d, e).

### Incorporation of VSV-G stimulated the endosomal release of PMVs

**Fig. 4:**
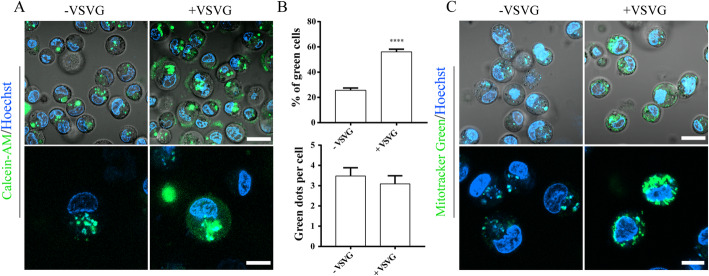
Phagocytosis and endosomal escape of PMVs. **a** PMVs were generated from hUCMSCs after incubation with Calcein-AM (green) for 30 min. Fusogenic VSV-G viral membrane glycoprotein was prepared from Ad293 cells after transfection with pLP-VSVG for 48 h. PMVs were incubated with or without VSV-G for 20 min and then added into HepG2 cell culture in 96-wells. Cells were stained with Hoechst (blue) at 24 h, harvested, and examined by confocal microscopy (upper panel, scale bar = 20 μm). An enlarged view of typical endosomal release is shown in the lower panel (scale bar = 10 μm). **b** Percentage of cells with fluorescence green signal in the cytoplasm (top, indicating endosomal escape of PMVs) and green dot per cell (bottom, number of PMVs being phagocytosed) are shown as mean ± SEM (*n* = 3 experiments). Student’s *t* test is performed between cultures with or without the addition of VSV-G. ***P < 0.001; **P < 0.01; *P < 0.05. **c** PMVs were generated from hUCMSCs after staining with MitoTracker-Green for 30 min. PMVs were incubated with or without VSV-G for 20 min and then added to HepG2 cell culture in 96-wells. Cells were stained with Hoechst (blue) at 24 h, harvested, and examined by confocal microscopy, scale bar = 10 μm

Viral membrane glycoprotein VSV-G is essential for membrane fusion and release of the viral genome into the host cytoplasm. We tested if the incorporation of VSV-G could improve the endosomal release of PMVs. To this end, hUCMSCs were stained with Calcein-AM before being used for PMV production. The result showed that VSV-G did not enhance phagocytosis of PMVs into HepG2 cells; however, the endosomal release of PMV content increased dramatically (Fig. 4a, b). To confirm the release of mitochondria, hUCMSCs were stained with MitoTracker-Green before PMV generation. The result clearly showed that mitochondria from hUCMSCs were efficiently delivered into HepG2 cells via PMVs (Fig. 4c).

### PMVs restored normal cellular functions in APAP-treated HepG2 cells

**Fig. 5:**
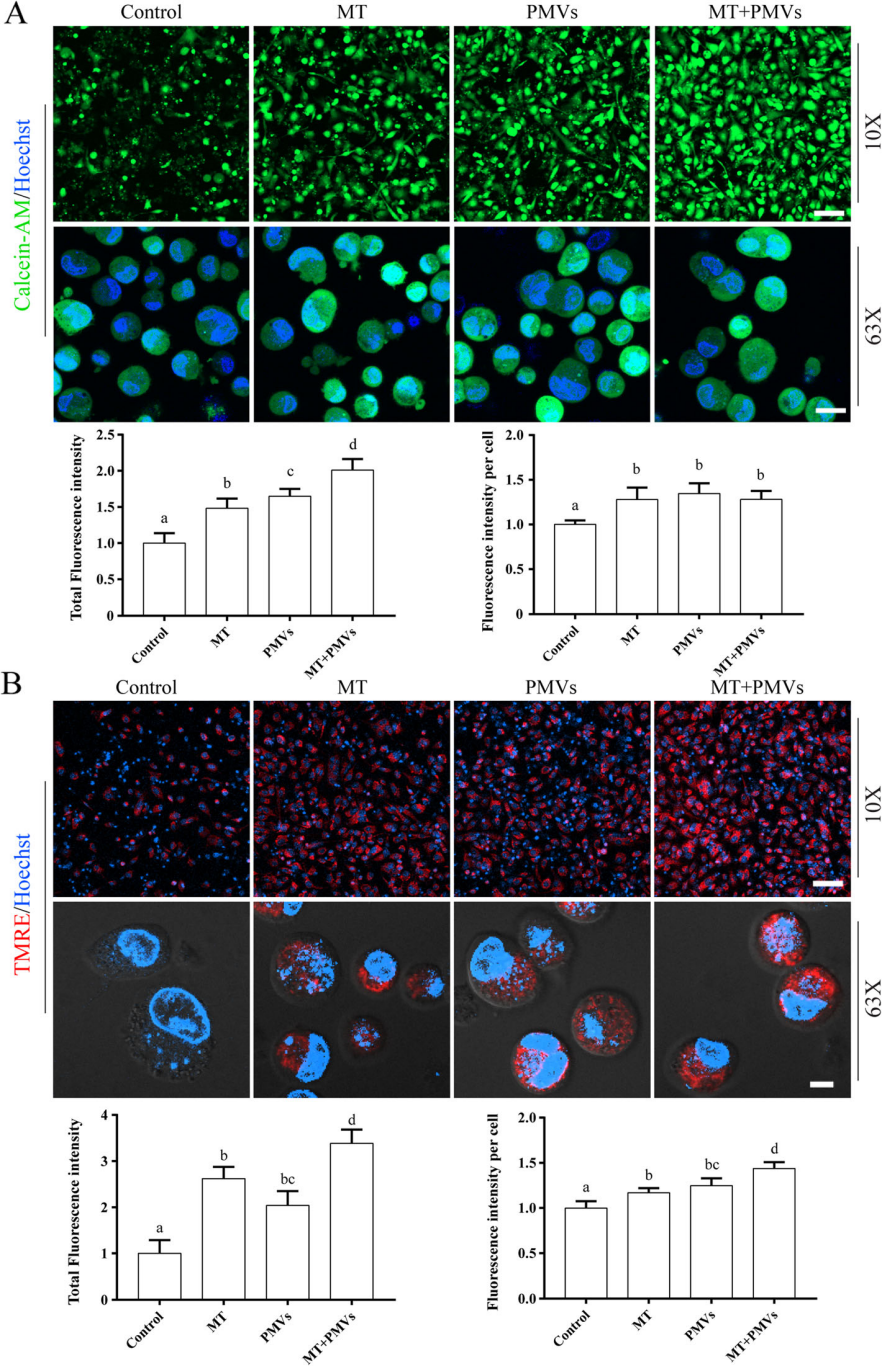
PMVs increase the viability of HepG2 cells treated with APAP*.* HepG2 were treated with 90 mM APAP for 3 h. After washing, cells were harvested and re-seeded in 96-wells precoated with ECM (extracellular matrix) prepared from HTB9 cells. Mito-Tempo (MT) or PMVs from hUCMSCs (with VSV-G) were added at 4 h. PBS was used as the negative control. **a** Cells were stained with Calcein-AM (green) and Hoechst (blue) at 48 h. Cells in the attachment (scale bar = 100 μm) and in suspension (scale bar = 20 μm) were analyzed by confocal microscopy with a × 10 or × 63 objective. Total fluorescence intensity and fluorescence intensity of individual cells are shown as mean ± SEM (*n* = 3 experiments). Bars sharing a letter in common are not significantly different (*P* > 0.05) (one-way ANOVA Student’s *t* test). **b** Cells were stained with TMRE (red) and Hoechst (blue) at 48 h. Cells in the attachment (scale bar = 100 μm) and in suspension (scale bar = 20 μm) were analyzed by confocal microscopy with a × 10 or × 63 objective. Total fluorescence intensity and fluorescence intensity of individual cells are shown as mean ± SEM (*n* = 3 experiments). Bars sharing a letter in common are not significantly different (*P* < 0.05) (one-way ANOVA Student’s *t* test)

hUCMSCs are excellent stem cell sources for the treatment of a variety of diseases, and the study tested whether PMVs of hUCMSCs could have similar therapeutic potential. HepG2 cells were subjected to a high concentration of APAP to mimic acute liver damage by APAP overdose. The result showed that the addition of PMVs significantly increased the viability of APAP-treated HepG2 cells compared to that of control (Fig. 5a). The effect of PMVs was comparable to that of Mito-Tempo (MT), which has been reported to be superior to *N*-acetylcysteine (NAC) in the treatment of APAP overdose. Eventually, a combination of PMVs and MT provided dramatic protection to HepG2 cells against APAP-induced damage.

APAP has been reported to cause mitochondrial damage [23]. We therefore evaluated mitochondrial membrane potential with TMRE staining. Similar to cell viability, the addition of PMVs reversed the decrease of mitochondrial membrane potential in APAP-treated HepG2 cells (Fig. 5b). The effect of PMVs was comparable to that of Mito-Tempo, and a combination of PMVs and Mito-Tempo led to even higher levels of mitochondrial membrane potential (Fig. 5b).

**Fig. 6:**
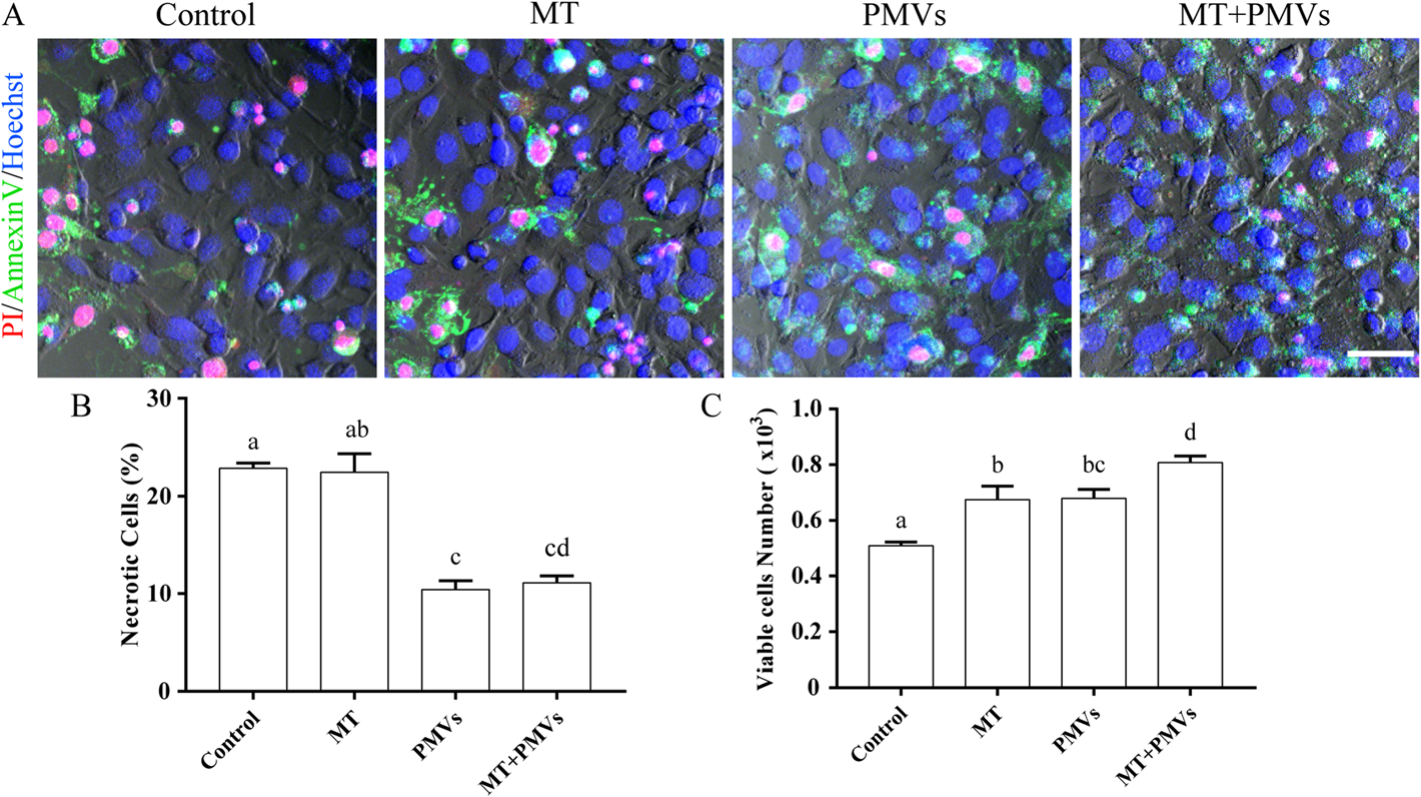
PMVs rescue HepG2 cells from APAP-induced necrotic cell death. HepG2 were treated with 90 mM APAP for 3 h. After washing, cells were harvested and re-seeded in 96-wells precoated with ECM (extracellular matrix) prepared from HTB9 cells. Mito-Tempo (MT) or PMVs from hUCMSCs (with VSV-G) were added at 4 h. PBS was used as the negative control. **a** Cells were stained with Annexin V-FITC (green), propidium iodide (red), and Hoechst (blue) at 48 h, and subsequently, cells in an attachment were analyzed by confocal microscopy, scale bar = 50 μm. **b**, **c** Percentage of necrotic cells and viable cell number are shown as mean ± SEM (*n* = 3 experiments). Bars sharing a letter in common are not significantly different (*P* > 0.05) (one-way ANOVA Student’s *t* test)

**Fig. 7:**
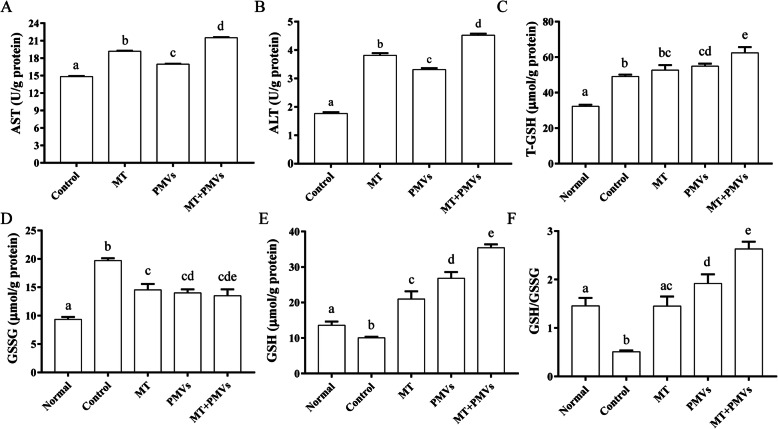
PMVs restored cellular physiology in APAP-treated HepG2 cells*.* HepG2 were treated with 90 mM APAP for 3 h. After washing, cells were harvested and re-seeded in 96-wells precoated with ECM (extracellular matrix) prepared from HTB9 cells. Mito-Tempo (MT) or PMVs from hUCMSCs (with VSV-G) were added at 4 h. PBS was used as the negative control. Intracellular levels of AST (**a**), ALT (**b**), total (T-) GSH (**c**), and GSSG (**d**) were measured using respective colorimetric approaches. GSH (**e**) and the ratio of GSH to GSSG (**f**) were calculated as described in the “Materials and methods” section. The values are shown as mean ± SEM (*n* = 3 experiments). Bars sharing a letter in common are not significantly different (*P* > 0.05) (one-way ANOVA Student’s *t* test)

We then carried out experiments to investigate whether PMVs were able to restore redox balance in APAP-treated HepG2 cells. The result showed that the cellular redox state in APAP-treated HepG2 cells returned to about normal levels after the addition of PMVs or Mito-Tempo, as evidenced by the increased amount of reduced glutathione (GSH) and therefore the ratio of reduced to oxidized GSH (GSH/GSSH) (Fig. 7c–f). In addition, PMVs increased significantly the cellular levels of aspartate aminotransferase (AST) and alanine aminotransferase (ALT) compared to that of control (Fig. 7a, b). Furthermore, HepG2 cells treated with APAP showed a dose- and time-dependent decrease in viability (Figure S1/2). However, the addition of PMVs significantly rescued HepG2 cells from APAP-induced necrotic cell death compared to that of control or addition of Mito-Tempo (Fig. 6).

## Discussion

The objective of this study was to evaluate whether PMVs could be exploited as a surrogate of hUCMSCs for stem cell therapy using APAP-treated HepG2 cells as a cellular model. The definition of hUCMSCs proposed by the International Society for Cell Therapy is the presence of surface molecules CD73, CD90, and CD105 while the absence of the differentiation markers CD14 or CD11b, CD19 or CD79a, CD34, CD45, and especially antigen presentation molecules such as HLA-DR [24], which is the primary cause underlying the immunologic tolerance of stem cell therapy. Flow cytometry analysis demonstrated that PMVs displayed specific characteristics that were identical to hUCMSCs in terms of cell surface markers (Fig. 1b, c), which was further confirmed by the analysis of the transcriptomic profile (Fig. 2), suggesting PMVs would be as immune unresponsive as hUCMSCs. Moreover, transcripts of stemness genes (GATA4/5/6, NANOG, OCT1/2/4A/4B) were similarly present in PMVs of hUCMSCs (Fig. 1d). Although not verified, these stemness genes could potentially rejuvenate target cells after being translated into functional proteins.

Besides the transcripts of stemness gene, PMVs of hUCMSCs also enclosed abundant mitochondria as well as other organelles such as lysosomes and endoplasmic reticulum (Fig. 3). When cells grow older and eventually turn into senescent, they accumulate a large amount of damaged and dysfunctional organelles, which have been implicated to play a causal role in the development of various aging diseases such as Alzheimer’s disease and lysosomal storage diseases [25, 26]. Organelles in PMVs of hUCMSCs are probably intact and functional. Therefore, PMVs of hUCMSCs could be an excellent source for regenerative medicine to replace impaired counterparts in target cells.

It is worthy to note that PMVs generated in the present study by a combined chemical and mechanical protocol have a larger and much more homogenous size compared with our previous study [19]. Cytochalasin B is an effective and reversible inhibitor of the actin filament, which has been routinely used for chemical enucleation for a variety of cells [27, 28]. In this study, enucleated cytoplasts were indeed generated after ultra-centrifugation; however, the majority of the hUCMSCs broke into smaller vesicles for unknown reasons. Nonetheless, the lack of polymerizing actin filament apparently facilitated the production of PMVs after further mechanical extrusion. Moreover, the study had found depolymerization of microtubules with colchicine further improved the enucleation efficiency [22]. Furthermore, the use of colchicine increased the number of mitochondria in PMVs (data not shown), which is consistent with the notion that mitochondria are associated with microtubules.

In the literature, several groups have reported the use of isolated mitochondria for the treatment of diseases linked to mitochondrial dysfunction [29, 30]. It needs to be emphasized that mitochondria usually exist as a network or in tubular form. The isolation procedure under a hypotonic condition not only leads to increased membrane permeability and reduced membrane potential, but also produces mitochondrial fragments in dot shape, which definitely mitigates the functionality. In contrast, mitochondria in PMVs of hUCMSCs reported in this study are clearly shown in a rod shape and with a normal membrane potential (Fig. 3). Eventually, high quality plus the integration of fusogenic VSV-G viral glycoprotein in PMVs ensured efficient delivery of mitochondria and other cellular components into HepG2 cells (Fig. 4).

Using the APAP-treated HepG2 cell model of acute liver injury, the study probed whether PMVs of hUCMSCs could provide beneficial effects as that of stem cell therapy. APAP has been reported to cause severe necrotic cell death in hepatocytes [31–33]. In consistent with this observation, the study found cell death but a negligible amount of Annexin V-positive while PI-negative HepG2 cells after APAP treatment (Figure S1/S2/6). The addition of PMVs significantly reduced the percentage of necrotic cells while increased the total number, cell viability, and mitochondrial membrane potential (Figs. 5 and 6). Likewise, PMVs increased significantly the cellular levels of AST, ALT, and GSH and attenuated the GSSG formation (Fig. 7), confirming that PMVs could have therapeutic potential as hUCMSCs. However, unlike traditional stem cell therapies [10, 34], PMVs probably functioned via transplanting healthy cytosol and organelles to remediate damaged cellular structures in target cells directly. In addition, several groups have reported the effects of MSC-derived exosomes in xenobiotic-induced liver injury models, including alleviation of acute liver injury and fibrosis in CCl_4_-treated mouse via suppression of oxidative stress and apoptosis [35], and increase of cell viability and reduction of ROS activity in APAP- or H_2_O_2_-treated HepG2 cells by exosome-rich fractionated secretome [36]. Compared with PMVs, exosome production is more time-consuming, while the yield is much lower since PMVs are micrometer in size. Furthermore, unlike PMVs, the ability to deliver cellular organelles has not been demonstrated with exosomes, which is essential for repairing or replacing damaged cellular structures.

Lastly, PMVs provide complementary and additive effects with Mito-Tempo in rescuing APAP-induced injury in HepG2 cells (Figs. 5, 6, and 7). Mitochondrial oxidative stress has been considered to be critical in the progression of APAP-induced hepatotoxicity [37]. Mitochondria-targeted antioxidant Mito-Tempo has been shown to be more effective compared to NAC or Tempo (antioxidants lacking a mitochondrial targeting signal) [33]. Furthermore, autophagy-inducing pharmaceuticals could protect hepatocytes via the removal of APAP protein adducts and damaged mitochondria [4, 38]. Results from other studies have also revealed that soluble protein aggregates could be removed by proteasomes via ubiquitination, while insoluble protein aggregates were removed by autophagy [39]. Although not directly tested, we proposed that PMVs might be able to stimulate cellular waste disposal processes via transferring lysosomes and proteasomes (Fig. 3).

In conclusion, the results suggested that antioxidant Mito-Tempo terminated the propagation of oxidative injury in APAP-treated HepG2 cells, and subsequently, PMVs rescued the cells via clearing damaged cellular components while providing functional organelles. The application of PMVs in vivo has not been formally investigated, and conceivably a myriad of hurdles needs to be overcome. Nonetheless, the PMVs of hUCMSCs present a novel approach to broaden the translational perspective of stem cell therapy.

## Conclusions

The results suggest that PMVs from hUCMSCs could be used as a novel stem cell therapy for the treatment of APAP-induced liver injury.

## Supplementary information

**Additional file 1.** Supplementary information and figures.

## Data Availability

The datasets generated and/or analyzed during the current study are available from the corresponding author on reasonable request. All data generated or analyzed during this study are included in this published article (and its supplementary information files).

## Abbreviations

APAP: Acetaminophen
ALF: Acute liver failure
ALT: Alanine transaminase
AST: Aspartate transaminase
DEGs: Differentially expressed genes
ER: Endoplasmic reticulum
ECM: Extracellular matrix
GSH: Glutathione
GSSG: Glutathione disulfide
HISAT: Hierarchical indexing for spliced alignment of transcripts
hUCMSCs: Human umbilical cord mesenchymal stem cells
MSCs: Mesenchymal stem cells
MT: Mito-Tempo
NAPQI: *N*-acetyl-p-benzoquinone imine
NAC: *N*-acetylcysteine
PBS: Phosphate-buffered solution
PMVs: Plasma membrane vesicle
ROS: Reactive oxygen species
RPKM: Reads per kilobase per million reads
VSV-G: Glycoprotein G of the vesicular stomatitis virus
