# Supplementary material for: Plasma membrane vesicles of human umbilical cord mesenchymal stem cells ameliorate acetaminophen-induced damage in HepG2 cells: a novel stem cell therapy
Source: Stem Cell Res Ther. 2020 Jun 8;11:225. doi: 10.1186/s13287-020-01738-z (PMC7278066; doi:10.1186/s13287-020-01738-z)
Supplement: Supplementary file 1 — Additional file 1. Supplementary information and figures. [file 13287_2020_1738_MOESM1_ESM.docx]

**Plasma membrane vesicles of human umbilical cord mesenchymal stem cells ameliorate acetaminophen-induced damage in HepG2 cells: A novel stem cell therapy**

Mei-jia Lin^1^, Shuang Li^1^, Lu-jun Yang^2^*, Dan-yan Ye^2^, Xin Zhang^3^, Ping-nan Sun^4^, Chi-ju Wei^1^*.

**Supplemental Materials**

**APAP-induced HepG2 cell death analysis**

About 2×10^5^ cells of HepG2 were treated with different doses (0, 10, 30, 50, 70 and 90 mM) APAP in 200 μl culture medium for 3 h, or treated with different times (0, 10 min, 2 and 3 h) with 90 mM APAP. The medium was then discarded and cells were harvested and stained with Annexin V-FITC (50 μg/ml), Propidium Iodide (100 μg/ml) and Hoechst (10 μg/ml) for 20 min, and subsequently analyzed by confocal microcopy with 10X and 63X objective.


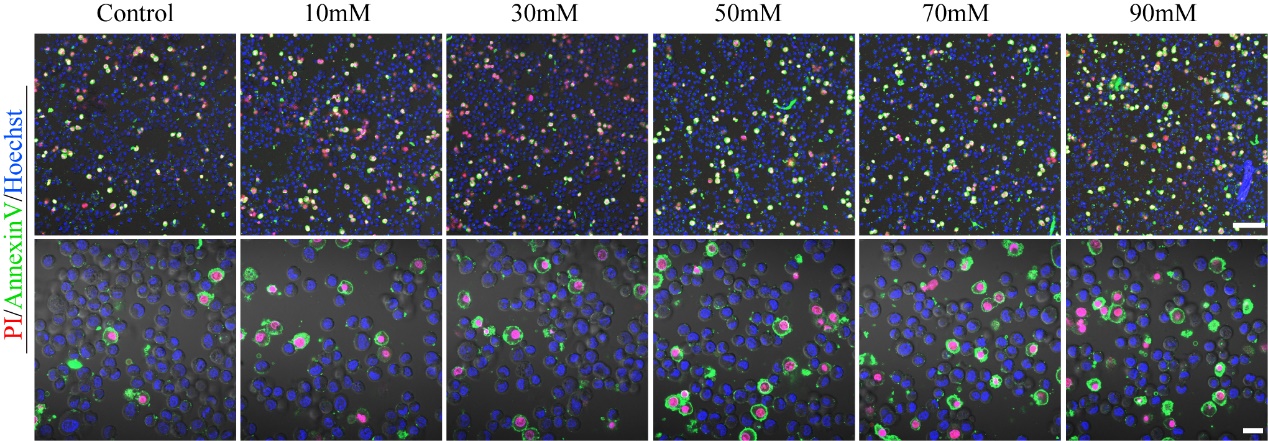


*Figure S1* *HepG2 cell death induced with different doses of APAP.*

HepG2 were treated with 0, 10, 30, 50, 70 and 90 mM of APAP for 3 h. After washing, cells were harvested and stained with Annexin V-FITC (Green), Propidium Iodide (Red) and Hoechst (Blue), and examined by confocal microscopy using 10X (Upper panel, Scale bar = 100 μm) and 63X (Lower panel, Scale bar = 20 μm) objective.


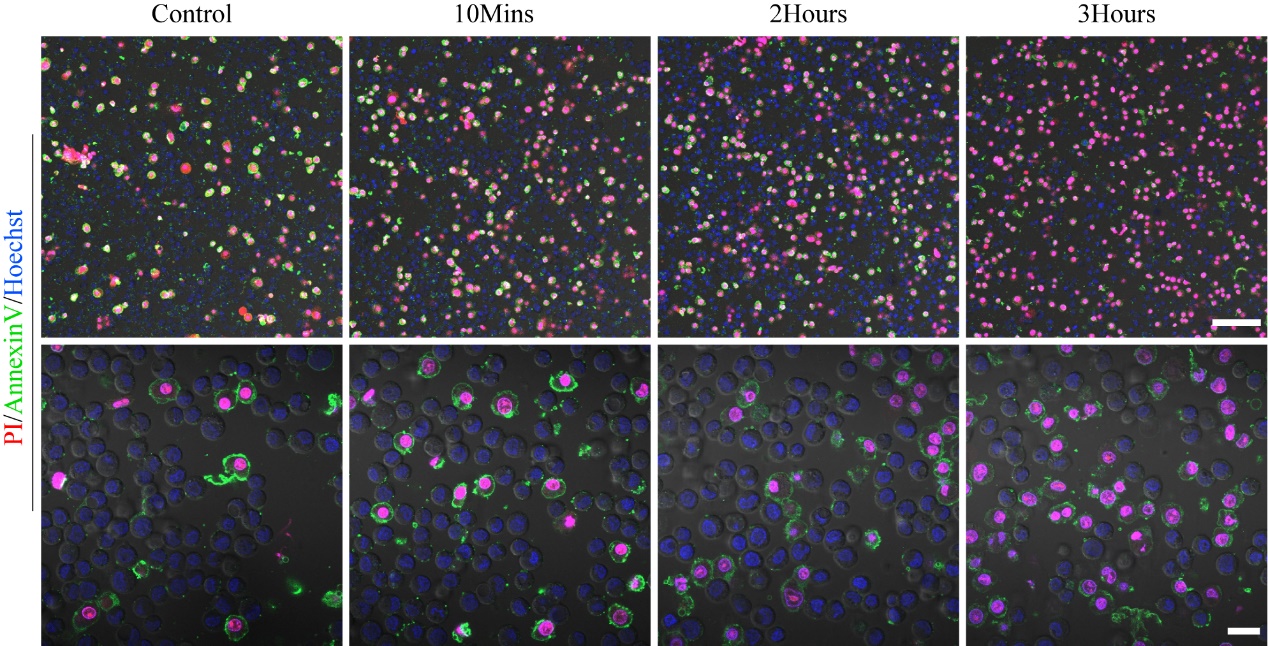


*Figure S2 A time-course study of HepG2 cell death treated with APAP.*

HepG2 were treated with 90 mM of APAP for 0, 10 min, 2 and 3 h. After washing, cells were harvested and stained with Annexin V-FITC (Green), Propidium Iodide (Red) and Hoechst (Blue), and examined by confocal microscopy using 10X (Upper panel, Scale bar = 100 μm) and 63X (Lower panel, Scale bar = 20 μm) objective.
